# Supplementary material for: Transcription modulates chromatin dynamics and locus configuration sampling
Source: Nat Struct Mol Biol. 2023 Aug 3;30(9):1275–85. doi: 10.1038/s41594-023-01059-8 (PMC10497412; doi:10.1038/s41594-023-01059-8)
Supplement: Supplementary file 1 — Contains Supplementary Notes and Supplementary Figure 1 [file 41594_2023_1059_MOESM1_ESM.pdf]

nature structural & molecular biology

Article

https://doi.org/10.1038/s41594-023-01059-8

Transcription modulates chromatin dynamics and locus configuration sampling

In the format provided by the authors and unedited

Transcription modulates chromatin dynamics and locus configuration sampling

Giada Forte[1], Adam Buckle[2], Shelagh Boyle[2], Davide Marenduzzo[1], Nick Gilbert[2,*], and Chris A. Brackley[1,*]

1. SUPA, School of Physics and Astronomy, University of Edinburgh, Peter Guthrie Tait Road, Edinburgh EH9 3FD, United Kingdom
2. MRC Human Genetics Unit, MRC Institute of Genetics & Cancer, University of Edinburgh, Western General Hospital, Edinburgh, EH4 2XU, UK
[*] Correspondence: Nick.Gilbert@ed.ac.uk or C.Brackley@ed.ac.uk

---

Supplementary Notes

Contents

1. Additional Simulation Methods
2. Simulation Input Data
3. CaptureC data analysis
4. Analysis of Simulation data
5. Alternative configurational dynamics analysis – enhancer-promoter collisions
6. Effect of varying loop extrusion parameters
7. Two models for simulating alpha amanitin treatment

1 Additional Simulation Methods

In the HiP-HoP model[1–5], a section of a chromatin fibre is represented as a chain of beads connected by springs. We then use molecular dynamics simulation methods to evolve the configuration of this polymer based on a set of phenomenological potentials which describe how the beads interact. Each bead in the chain represents a 1 kbp region of the genome. Three additional model ingredients drive the chromatin configuration: interactions with spheres representing complexes of proteins[6–8], a heteromorphic polymer structure[9], and loop extrusion[10,11].

1.1 Protein bridges and the polymer model

Interactions among polymer beads are defined by a force field consisting of four potentials. First, non-adjacent beads interact sterically via a Week-Chandler-Anderson (WCA) potential given by

$$V_{\text{WCA}}(r_{i,j}) = 4k_BT\left[\left(\frac{\sigma}{r_{i,j}}\right)^{12} - \left(\frac{\sigma}{r_{i,j}}\right)^{6} + \frac{1}{4}\right]\Theta(2^{1/6}\sigma - r_{i,j}), \tag{1}$$

where $r_{i,j} = |\mathbf{r}_i - \mathbf{r}_j|$ is the separation of beads i and j located at positions $\mathbf{r}_i$ and $\mathbf{r}_j$ respectively, T and k_B are the temperature of the system and the Boltzmann constant, σ is the bead diameter, and $\Theta(x)$ is the Heaviside function [$\Theta(x) = 1$ if $x > 0$, or $\Theta(x) = 0$ otherwise]. Second, adjacent beads are connected by finitely-extensible non-linear elastic (FENE) springs with interaction energy defined by

$$V_{\text{FENE}}(r_{i,i+1}) = -\frac{K_{\text{FENE}}}{2}R_0^2 \ln\left[1 - \left(\frac{r_{i,i+1}}{R_0}\right)^2\right] + V_{\text{WCA}}(r_{i,i+1}),$$

with $K_{\text{FENE}} = 30k_BT/\sigma^2$ being the bond strength and $R_0 = 1.6\sigma$ the maximum bond length. Third, the polymer is afforded a finite bending stiffness via a Kratky-Porod potential given by

$$V_{\text{BEND}}(\phi_i) = K_{\text{BEND}}\left(1 + \cos\phi_i\right),$$

where ϕ_i is the angle between beads $i-1$,i and $i+1$, while $K_{\text{BEND}} = 4k_BT$ leads to a persistence length comparable to that of chromatin. Finally, additional springs are used to "crumple" the polymer in some regions (i.e. to give it heteromorphic properties). A harmonic spring potential is used, described by

$$V_{\text{HARM}} = K_{\text{HARM}}(r - R_{\text{HARM}})^2,$$

with spring constant $K_{\text{HARM}} = 200\varepsilon/\sigma^2$ and bond length $R_{\text{HARM}} = 1.1\sigma$. This potential is applied between next-nearest neighbour beads (i and $i+2$) which do not present ChIP-seq peaks for acetylation of histone H3 in lysine 27 (H3K27ac) which usually identifies open chromatin regions.

Protein complexes are represented by spheres of the same size as the polymer beads. They interact sterically with each other via the WCA potential as given in Eq. (1). Proteins have a strong attractive interaction with a subset of polymer beads which represent protein binding sites on the chromatin, a weak attractive interaction with chromatin beads marked by H3K27ac, and a steric (WCA) interaction with other chromatin beads. The attractive interactions are described by a shifted and truncated Lennard-Jones potential

$$V_{\mathrm{LJ/cut}}(r_{i,j}) = [V_{\mathrm{LJ}}(r_{i,j}) - V_{\mathrm{LJ}}(r_c)]\,\Theta(r_c - r_{i,j}),$$

with

$$V_{\mathrm{LJ}}(r) = 4\varepsilon\left[\left(\frac{\sigma}{r}\right)^{12} - \left(\frac{\sigma}{r}\right)^{6}\right],$$

where $r_c = 1.8\sigma$ is the cutoff distance and ε is the interaction strength. For strong interactions (i.e. with protein binding sites) we use $\varepsilon = 8k_BT$, while for weak non-specific interactions we use $\varepsilon = 2k_BT$.

During the simulations proteins switch back and forward between a binding and a non-binding state with at rate k_{switch} [8]. This represents post-translation modifications which alter the protein-DNA binding affinity (e.g. phosphorylation). When in the non-binding state the interaction between proteins and chromatin binding sites revert to the WCA potential.

1.2 Langevin Dynamics

We use the LAMMPS molecular dynamics software to perform the simulations [12]. Proteins and polymer beads move according to the Langevin equation

$$m_i \frac{d^2\mathbf{r}_i}{dt^2} = -\nabla_i U - \gamma_i \frac{d\mathbf{r}_i}{dt} + \sqrt{2k_BT\gamma_i}\,\boldsymbol{\eta}_i(t), \tag{2}$$

where m_i is the bead mass, $\mathbf{r}_i$ is its position, U is the total potential energy of the system and γ_i is the friction due to an implicit solvent. The final term provides thermal noise where components of the vector $\boldsymbol{\eta}_i$ are such that

$$\langle \eta_{i,\alpha}(t)\rangle = 0 \text{ and } \langle \eta_{i,\alpha}(t)\,\eta_{j,\beta}(t')\rangle = \delta_{i,j}\delta_{\alpha,\beta}\delta(t-t'),$$

where $\delta_{i,j}$ is the Kronecker delta and $\delta(t-t')$ is the Dirac delta function.

In LAMMPS, Eq. (2) is solved using a velocity-Verlet scheme. We use a time step $\mathrm{dt} = 0.01\ \tau$, where τ is the simulation time unit (see below for mapping to real times).

1.3 Loop extrusion

The HiP-HoP model includes the loop extrusion mechanism thought to be performed by the cohesin complex [10,11,13]. Cohesin rings are represented by additional spring bonds between beads; the pair of beads to which the bond is applied is then moved outwards, i.e., from $(i, i+3) \to (i-1, i+4) \to (i-2, i+5)$ etc., to extrude a loop. Extruders are added at random $i, i+3$ positions with rate k_{on} and removed with rate k_{off}. Their positions are stepped stochastically at rate k_{ex}. The spring potential is given by

$$U_{\mathrm{EXTR}}(r_{i,j}) = U_{\mathrm{WCA}}(r_{i,j}) + K_{\mathrm{EXTR}}(r_{i,j} - r_0)^2,$$

where $K_{\mathrm{EXTR}} = 40k_BT/\sigma^2$ is the bond strength and $r_0 = 1.5\sigma$ is the bond length. An extruder is halted either when it meets another extruder, or when it meets a CTCF site whose direction is opposite to the extrusion direction. If an extruder is halted on one side, the position at the other side continues to be moved, extruding the loop. With this scheme, which is similar to that in, e.g. Ref. 10, there is no feedback from the 3D dynamics onto the extruder dynamics; for this reason, the positions of the CTCF sites in each simulation are the only thing affecting the extruder dynamics.

1.4 Simulation Setup

As detailed in the main text, in order to perform simulations in a realistic chromatin density, we consider a larger chromatin fragment than in Ref. 1. Specifically we simulate a 40,000 bead polymer representing a 40 Mbp chromatin fragment, in a square system of side 91 σ (with periodic boundary conditions). This gives a chromatin density of $\sim 50\,bp/\sigma^3$, which once mapped to real units is the same order of magnitude as *in vivo*. To be able to obtain a useful simulation data set, instead of simulating a 40 Mbp region around *Pax6*, we simulated a 3 Mbp region, and positioned 10 copies of this region along the 40,000 bead polymer (with a 910 kbp region of "unmarked" chromatin between each; since this is larger than the typical size of chromatin domains, we reasoned that it would be sufficient to isolate neighbouring locus copies). This means that a single simulation gives data equivalent to measuring 10 single cells. The limitation of this approach is that a given copy of the locus is embedded in an unrealistic background; comparison with experimental data (CaptureC and FISH, Supplementary Figs. 1,2) confirmed that this does not greatly affect predictions for interactions within the locus or locus configurations. To ensure that each copy of the locus was embedded in a similar background, in a given simulation we included a mixture of *Pax6* loci from the different cell lines, and in repeat simulations we randomly shuffled the positions of the loci from different cell lines. For example, this setup ensured that there were roughly the same number of proteins per binding site in all simulations (there are different numbers of binding sites within the locus in the different cell lines, so having a similar mixture of cell lines in each simulation preserves this ratio).

Results were then obtained by combining measurements for all copies of the locus in the same cell line. The majority of results presented are obtained from six independent simulations, giving in total 20 copies of the locus in each cell line. For results in Fig. 4h-m and Supplementary Fig. 4a, where we used different parameters, three independent simulations were performed (10 copies of the locus in each cell line).

As an initial condition, we arranged the 40,000-bead chains in a mitotic-like structure similar to those in Ref. 14. This was first relaxed in the absence of protein interactions but with loop extrusion. This allowed the chain to move away from its initial structure, but maintained a fractal globule-like probability of interaction as a function of genomic separation[15]. A further relaxation simulation was then done with the additional springs which "crumple" the polymer as detailed above.

In the majority of cases, each independent simulation was then run for a total time of $1.5 \times 10^6\,\tau$; we discarded the first $7 \times 10^5\,\tau$ to ensure that the system had reached a configuration representative of the *in vivo* locus and had reached a steady state. That is to say, that the global properties had reached a state where they fluctuated around a constant mean value (specifically, we checked that the radius of gyration of any given copy of the *Pax6* locus, or the radius of gyration of the entire polymer was not systematically changing in time). The presented results were obtained from the remaining $8 \times 10^5\,\tau$. For results in Figs. 4h-m and Supplementary Fig. 4a, where we used different parameters, shorter simulations of duration $5 \times 10^5\,\tau$ were used. When examining trajectories, the configurations at two time points separated by a short interval will be virtually identical; by examining the autocorrelation functions of measured quantities, we can calculate the typical time scale over which they change. From our observations, we determined that extracting a configuration every $2 \times 10^3\,\tau$ would give a representative trajectory; this means that a simulation of duration $8 \times 10^5\,\tau$ yielded 400 individual configurations.

1.5 Lengths, times and other units in simulations

In our simulations lengths are given in multiples of the polymer bead diameter σ; as detailed in the Methods section in the main text, we determined how this maps to a real length scale by comparison between simulated and experimental FISH measurements. (as in Ref. 1). For each pair of FISH probes in each cell line, we obtained a distribution of separations, and compared simulated and experimental distributions using the Kolmogorov-Smirnov statistic K_S. We use a mapping between simulation and physics units which minimises the mean value of K_S obtained across all pairs of probes in all simulated cell lines. This leads to $\sigma =$ 17.6 nm.

To map the simulation time unit τ to a physical time we compare our simulations to *in vivo* measurements of chromatin dynamics. Specifically, we measure the polymer bead mean squared displacement as a function of lag time [MSD(t)] averaged over all beads in all simulations. We then use a mapping between simulation and physical units which gives the best fit to MSD(t) curves obtained from Ref. 16, in which the authors performed particle tracking of probes at different chromatin regions in yeast. This leads to a value $\tau \approx 2.07 \times 10^{-3}$ s. The total run time of a simulation (after removing the first $7 \times 10^5\,\tau$ as detailed above) is therefore equivalent to about 27 minutes. We use the yeast data set as it provides MSD(t) curves over a very broad range of lag times (0.02–400 s) for seven chromosome loci. Similar data sets are available for mammalian cells[17–19], though these cover shorter lag times, are not as comprehensive, and involve several different imaging and tracking methods. A range of different effective diffusion constants and anomalous diffusion exponents have been reported; for example, using a mapping based on tracking of a chromosome locus in the nuclear interior of HeLa cells[17] leads to a simulation duration of 16 minutes, data from mouse Hep1-6 cells[18] leads to a 25 minute duration, while data from a human breast cancer cell line[19] leads to a simulation time of around 90 minutes. This highlights that our comparisons to real time scales are approximate, but different mappings give the same order of magnitude; this does not affect comparisons between different simulation measurements.

Energies are given in units of k_BT, and for simplicity we set the mass of all components (chromatin beads and proteins) to be the same ($m_i = m$ for all i). For computational efficiency we set $\gamma_i = 2m/\tau$; this is an approximation which means that the components have more inertia than in reality, but it renders the simulations much more efficient. This enables longer simulations to be performed such that we can assess the dynamics on relevant time scale (minutes).

As detailed above, we performed simulations of $L = 40,000$ bead long polymers containing 10 copies of the *Pax6* locus. For the majority of simulations we included 1000 proteins, and these switch back and forth between an active and inactive state at a rate $k_{\mathrm{sw}} = 10^{-3}\,\tau^{-1}$; using the above mapping this means proteins switch on a time scale of $0.48\,s$. In Figs. 4h-m and Supplementary Fig. 4a we present results from simulations using different values for these parameters as indicated in the relevant captions. Other important rates are related to loop extrusion and we used values: $k_{\mathrm{on}} = 2 \times 10^{-2}\,\tau^{-1}$, $k_{\mathrm{off}} = 2.5 \times 10^{-5}\,\tau^{-1}$, and $k_{\mathrm{ex}} = 2\,\mathrm{bp}/\tau$. Our choice of parameters were based on those in Ref. 1, which were previously found to lead to good predictions of locus configurations. Due to the changes in the simulation set up, it was not possible to exactly preserve all parameter values.

2 Simulation Input Data

We use four sets of genomic data as an input to the simulations. Specifically, ATAC-seq data were used to identify active protein binding sites (we assumed all accessible DNA regions are binding sites); ChIP-on-chip data for H3K27ac were used to identify open chromatin regions; and ChIP-on-chip data for CTCF and the cohesin sub-unit Rad21 were used to identify loop anchor sites. We considered a 3 Mbp region around *Pax6* (chr2:104,000,000-107,000,000 mm9 genome build), mapping all features to beads representing 1 kbp of chromatin (i.e., this sets the resolution of the model at 1 kbp)

We previously obtained ATAC-seq data from each of the three cell lines as reported in Ref. 1 (available at GEO:GSE119656).

As detailed in that reference, Nextera adaptors were trimmed from sequenced reads using the ‘trim galore’ utility, and aligned to the mm9 genome using bowtie2[20]. Read pile-ups were generated and corrected for read depth using the ‘genome coverage’ tool from the Bedtools suite[21]. Peak calling was performed using MACS version 2.1.1[22], with a q-value cut off of 0.05. In the model protein binding sites were identified by mapping the centre of each ATAC-seq peak to the corresponding 1 kbp polymer bead.

ChIP-on-chip data for H3K27ac, CTCF, and Rad21 were previously obtained from each of the three cell lines, again from Ref. 1 (available at GEO:GSE119659, GSE119658 and GSE120665). As detailed in that reference, the Bioconductor tool “Ringo” was used for pre-processing, normalisation, combining replicates and peak calling[23].

To identify “open chromatin” regions, we mapped the H3K27ac data to the set of polymer beads, and identified all beads which had an overlapping ChIP peak. For these beads we then did not include the additional springs which act to crumple the polymer.

To identify loop anchor sites we used the set of CTCF peaks which overlap with Rad21 peaks. We then found the directionality of each site by locating its underlying binding motif. The binding motif was obtained from the JASPAR database (matrix MA0139.1[24]); the FIMO tool from the MEME suite[25] was used to search for and identify the direction of motifs within each peak. A given CTCF peak can contain multiple binding motifs, therefore a site’s directionality was identified as the orientation of the motif with the highest score in terms of its match to the consensus motif. If there were motifs with similar score with different orientation we identified the peak as having both orientations. Peaks which did not overlap a CTCF motif were excluded. In each repeated simulation and copy of the locus we included only a subset of CTCF peaks, choosing them stochastically with a probability based on the peak height; this takes into account cell-to-cell variability in the occupancy of different CTCF sites. Finally, as for ATAC-seq peaks, we mapped the centre of the peak to a specific polymer bead. Note that within this scheme the binding and unbinding dynamics for CTCF are not fully captured, and effective CTCF residence times are likely overestimated[26]; however, the loop extrusion dynamics are mainly dependent on the extrusion and extruder unbinding rates[10], so this is unlikely to affect our results.

3 CaptureC data analysis

We previously generated CaptureC data for *Pax6* HIGH, ON and OFF cells[1] (data available at GEO:GSE120666). For this study we generated new CaptureC data for *Pax6* HIGH cells which had been treated with alpha amanitin or bleomycin as detailed in the Methods section in the main text. Oligonucleotides were designed to target restriction enzyme fragments at 13 locations across the locus as detailed in Supplementary Table 1.

For all CaptureC experiments we analysed the data using the capC-MAP software[27] (which uses bowtie[28], samtools[29], and cutadapt[30] software). In brief, first reads were checked for adaptor sequence and this was removed using the cutadapt tool[30]. An *in silico* restriction enzyme digestion was performed, breaking reads at the *DpnII* cutting sequence (GATC). The resulting read fragments were mapped to the mouse genome (mm9 build) using bowtie[28]. Aligned reads were filtered for PCR duplicates, and checked against the set of “target” restriction enzyme fragments for which oligonucleotide probes were designed. Only read pairs which identified a ligation event between a target and a (uniquely identifiable) “reporter” (i.e. non-target) fragment were retained. CaptureC interaction profiles were obtained for each target and sliding window binning and smoothing applied (using a window of 6 kbp and a bin width of 3 kbp; see Ref. 27 for details). Replicate data sets were compared, checked for consistence, and then combined to produce the final interaction profiles.

4 Analysis of Simulation data

4.1 Simulated CaptureC profiles

Simulated CaptureC interaction profiles were obtained from locus conformation generated from the HiP-HoP model following the same procedure as in Ref. 1. Specifically, we extract configurations from the simulations at $2000\,\tau$ intervals, and sample interactions stochastically. For a given viewpoint/target, for each copy of the locus in a simulation we select a bead at random from within the target region, and select a second bead at random from within the rest of that copy of the locus. We then accept this as an interaction with probability $P(x) = \exp(-x^2/x_0^2)$, where x is the 3D separation of the two beads, and x_0 is a threshold set at $x_0 = 3.5\sigma$; this is repeated L times where $L = 3000$ is the number of beads in that copy of the locus. We do this for each copy of the locus from a given cell type in each extracted configuration. We then repeat the whole procedure until the total number of accepted interactions is at least the same as than the number of reads typically obtained for a single viewpoint in our CaptureC data. From this procedure we generate a simulated interaction profile. Finally, we scale the profile such that the average number of reads per bead is the same in simulations and experiments.

4.2 Quantitative comparison between simulation and fixed-cell data

To quantitatively compare simulations with Capture-C and FISH data we followed the same procedure as in Ref. 1, defining two metrics, or ‘scores’.

The Q-score quantifies how well a set of simulated Capture-C interaction profiles agrees with experimental data[1,7]. We took the set of scaled interaction profiles generated as detailed above, truncate values lower than 0.35 to zero, and used a sliding averaging window to smooth both the simulation and experimental data, before applying a peak finding algorithm to identify interactions

(the “findpeaks” function in the MATLAB software[31]). We used the peak positions and widths (but not heights) to test whether peaks in each data set overlap. The quantity

$$q_{ij} = \frac{n_{se} + n_{es}}{n_s + n_e},$$

for the ith viewpoint in cell type j can then be calculated; here n_s and n_e are the number of peaks found in the simulation and experimental data respectively, n_{se} is the number of peaks in the simulation data which overlap with one or more peaks in the experimental data, and n_{es} is the number of peaks in the experimental data which overlap with one or more peaks in the simulation data. The overall Q-score is then the average over all viewpoints and cell types. To put the values of the score in context, we generated a randomised control data set by assuming that the same number of Capture-C peaks as generated by the HiP-HoP model were randomly scattered within the locus (a peak at the viewpoint was assumed to always be present). We then averaged the Q-score generated by many realisations of the random peak selection.

The K-score quantifies how well distributions of separations between probes within the simulated locus compare to FISH measurements[1]. We considered 18 probe pair separation distributions across the 3 cell lines, and the value of the simulation length unit which best fits the data was identified. The normalised two-sample Kolmogorov-Smirnov statistic describing the distance between each experimental and simulated distribution was then found. This statistic can have values between 0 (when the distributions are identical) and 1 (when there is no overlap). The overall K-score is the average of the normalised distances from all 18 probe pairs, subtracted from 1 (such that it takes a value 1 when the simulations are in complete agreement with the data). To provide a control, we generated a random simulated FISH data set (probe separations were chosen from a uniform distribution with the same maximum and minimum values as found in simulations).

4.3 Mean squared displacement and mobility of simulated FISH probes

In order to calculate the mean squared displacement (MSD) for a given simulated FISH probe, we find the centre of mass of all beads covered by that probe in a given copy of the locus

$$\mathbf{r}_{\text{CoM}} = \frac{1}{N}\sum_i \mathbf{r}_i,$$

where the sum runs over the N beads covered by the probe. In this way we obtain the trajectory $\mathbf{r}_{\text{CoM}}(t)$ and its MSD is calculated as

$$\text{MSD}(t) = \langle [\mathbf{r}_{\text{CoM}}(t+t_0) - \mathbf{r}_{\text{CoM}}(t_0)]^2 \rangle,$$

where angle brackets denote an average over times t_0, and t is the lag time. A MSD is obtained for each copy of the locus in each repeat simulation; in figures we plot the mean MSD over locus copies, and the standard error of this mean. Since we have 20 copies of the locus in each cell line (spread across 6 simulations), we have 20 independent MSDs, so the standard error is given by the standard deviation scaled by $1/\sqrt{20}$.

We define the mobility of a chromatin bead as the MSD at a fixed lag time t^*, specifically $\mathscr{M} = \text{MSD}(t^*)$, and the mobility profile is obtained by calculating $\mathscr{M}$ for each polymer bead. We used a lag time $t^* = 10^4\,\tau$ (approximately equivalent to 20.7 s) which is short enough to give good statistics (i.e., small errors). We take the mean over t_0 and locus copies, and the error is given by the standard error in the mean (shown as a shaded region around the lines in Fig. 1E in the main text). We also considered mobility profiles using different lag times $t^* = 2\times10^3\,\tau$ and $t^* = 2\times10^4\,\tau$; while this shifted the mobility profile up or down, it did not significantly change its shape. A mobility can also be calculated for a probe covering multiple chromatin beads by using the MSD for the centre of mass of those beads (Supplementary Fig. 4c).

For an object freely diffusing in 3D, the mean squared displacement will grow linearly with time, $\text{MSD}(t) = 6Dt$, where D is the diffusion constant. More generally, $\text{MSD}(t) \sim t^{\alpha}$; if the exponent $\alpha < 1$, the motion is said to be sub-diffusive. From the Rouse model for polymer dynamics[32] we expect the motion of a segment of a polymer to show an exponent $\alpha \approx 0.5$ (returning to diffusive behaviour at long lag times due to the polymer centre of mass motion). The inset in Fig. 2d reveals that the 40 kbp probe motion does show an exponent close to 0.5, at least up to lag times of about 250 s. A plateau in an MSD at long times is indicative of confined or constrained motion; given that our model includes several mechanisms which might constrain the motion we would expect a complicated MSD curve which goes through multiple regimes at long times.

4.4 Local density and extruder density measure

To generate the local density profiles as shown in Fig. 2e, we calculated the density ρ_i in the vicinity of chromatin bead i. This was defined as the total number of beads (polymer beads and proteins) within a sphere of radius R^* centred at $\mathbf{r}_i$, divided by the sphere volume. We used $R^* = 3\sigma$, which is the same value used as the ‘contact threshold’ for generating simulated CaptureC profiles as detailed above. Finally, we average over the 20 copies of the locus for each cell line, and calculate the standard error in the mean (shown as a shaded region around the line in Fig. 2e in the main text; this is often of a similar thickness to the line).

We calculate the extruder density measure ϕ_e by counting, for chromatin bead i, the number of configurations in a simulation where there is an extruder spring at beads $i-3$ to $i+3$ (i.e. within a distance 3σ from bead i). This is divided by the total number of considered configurations. We then average over all 20 copies of the locus of a given cell type, and calculate a standard error

in the mean. Profiles of ϕ_e across the locus are shown in Supplementary Fig. 5, with shaded regions around the lines showing the error. All ϕ_e values were used to generate the plot in Fig. 2i.

4.5 Correlations between quantities

In Fig. 2g we show the relationship between the mobility and density of chromatin beads. Each point represents a single bead and data are included for all three cell lines, with the two quantities being calculated as detailed above. Similar scatter plots are shown in Supplementary Fig. 4a, but the same quantity is compared between two sets of simulations with different parameters.

In Figs. 2i we show the correlation between mobility and H3K27ac density. Here the data are given for 20 kbp (20 bead) windows rather than individual beads (we consider a window around each bead, from bead 10 to bead 2990 within each 3000 bead copy of the locus). The mobility value for a given window is taken to be the mean value over the 20 beads. The H3K27ac density is defined as the fraction of the 20 beads which have that property. In Fig. 2i we show the correlation between mobility and extruder density; here the values used are for each bead and are calculated as detailed above. In both panels data are used from all 20 copies of all three cell lines.

4.6 Localness of interactions measure

To define a measure of the localness of interactions, for each chromatin bead we count interactions with other beads across the entire polymer. In this way, interactions with other locus copies are also included, meaning that these can be with chromatin regions further away than 3 Mbp (the size of the region around *Pax6* which we simulate). Since the surrounding chromatin environment in which the 3 Mbp locus is embedded in simulations is not representative of the *in vivo* environment, this measure should be considered only as a rough indication of interactions. Here, an interaction is defined as when two beads are closer together than 3.5σ in 3D space. We first exclude all interactions between beads separated by less than 5 kbp and more than 10 Mbp. Then the localness measure for a given bead within the locus is defined as the number of interactions with beads closer than (or equal to) 100 kbp away, divided by the number of interactions with beads further than 100 kbp away (Fig. 3a). This threshold distance is chosen such that interactions between promoters and distal enhancers will typically be counted as 'long ranged'. As with the other measures, we average over all copies of the locus for a given cell type. The error is the error in the mean, and in Fig. 3b this is shown as a shaded region around the lines (but is a similar size as the line thickness). In Fig. 3c values for mobility and localness for individual beads are used, and we include from all beads within all copies of the locus for all three cell types (beads in the 'spacer regions' between locus copies are not included).

4.7 Dynamics of the locus structure

To characterise the locus structure we defined a vector

$$\mathbf{X} = (x_{\mathrm{UP}}, x_{\mathrm{UD}}, x_{\mathrm{PD}}),$$

where x_{UP}, x_{UD}, and x_{PD} are the separations of the centre of mass of the URR and *Pax6*, URR and DRR, and *Pax6* and DRR FISH probe regions respectively. In this way $\mathbf{X}$ is a vector in a three-dimensional configuration space, and we can track its trajectory in time $\mathbf{X}(t)$ (Figs. 4a-e). The $\mathbf{X}$ vector can also be obtained for a single fixed cell in 3-colour FISH experiments.

The 'shape-change' parameter is then defined as the mean squared displacement of $\mathbf{X}(t)$

$$S(t) = \langle (\mathbf{X}(t+t_0) - \mathbf{X}(t_0))^2 \rangle,$$

where t is the lag time, and the angled brackets denote an average over trajectories and times t_0 in the same way as the mean squared displacement calculation detailed above.

A measure of the mean size of the locus at time t is given by the root mean squared of the vector $\sqrt{\langle \mathbf{X}(t) \rangle}$, where the angle brackets denote an average over time points and repeat simulations of the locus (Supplementary Fig. 2d left shows the distribution of locus size over all configurations in all copies of the locus for a given cell type). This same measure can be obtained for a single fixed cell from FISH experiments (distributions are show in Supplementary Fig. 2c left).

A measure of the variability of the locus configuration can be obtained from the volume of cloud of points in configuration space (Fig. 4d, all points along all of the trajectories from each copy of the locus for a given cell line are included). To estimate this we calculate the radius of gyration of of the points; this leads to

$$\text{variability} = \left(\frac{1}{NM} \sum (\mathbf{X} - \langle \mathbf{X} \rangle)^2 \right)^{3/2},$$

where the sum runs over the N points obtained from each of M repeat simulations, and the $3/2$ power ensures units of volume. The same measure can be made using $\mathbf{X}$ vectors obtained from single fixed cells from FISH experiments (Supplementary Fig. 2c right). Note that this is only a rough measure of volume, as it will depend on the distribution of points within the configuration space; since these points appear to be distributed largely homogeneously, we believe that it is nevertheless an informative measure.

5 Alternative configurational dynamics analysis – enhancer-promoter collisions

As noted in the main text, a complementary approach to characterise locus configurations is to examine specific promoter-enhancer interactions, and track these in time; e.g., the duration of a promoter-enhancer interaction (or 'collision') and the time interval between such interactions can be measured.

Results from an analysis considering the collision interval and interaction duration for interactions between the *Pax6* promoters and the two enhancers (URR and DRR) are shown in Supplementary Fig. 1. For each of these elements there were several ATAC-seq peaks in close proximity, so we considered a region at each element which covered all of the peaks. Specifically at the URR we considered a 13 chromatin bead region (chr2:105,444,500-105,457,500), at *Pax6* we considered an 11 bead region (chr2:105,509,500-105,520,500), and at the DRR an 11 bead region (chr2:105,629,500-105,640,500 all coordinates mm9 reference genome). A collision was defined as when the separation of the centre of mass of two regions is below $6\sigma \approx 106$ nm. This threshold was chosen as it is roughly equal to the radius of gyration of one of the 11 bead regions. Note this is a coarser measurement than the precise interaction profiles in the simulated CaptureC. From a given trajectory, the duration of periods where two elements are together were recorded as 'interaction durations', and the duration of periods where two elements are apart were recorded as 'collision intervals' (Supplementary Fig. 1a). Polymer configurations were only retained at intervals of $2 \times 10^3 \tau$, so this limits the temporal accuracy of these measurements.

Supplementary Fig. 1b shows the mean collision interval for *Pax6* and each of the enhancer regions (an average across all 20 trajectories for each cell line is shown). For a long uniform polymer, the mean time between collisions of any two internal points only depends on their separation. All three of the main model ingredients will alter this. Loop extruders can actively bring different polymer regions together, and so the collision interval will depend on the pattern of CTCF sites (loop anchors). The collision interval for a given pair of sites will also depend on the protein mediated stabilisation of other nearby loops, so binding site positions across the whole locus play a role, and so does the heteromorphic polymer structure between the pairs of sites, which alters the chromatin flexibility locally. All of these factors differ between the cell lines. In general, collision intervals are largest in *Pax6* OFF cells, where there are few protein binding sites or H3K27ac (open) regions. The *Pax6*-URR collision interval is similar in *Pax6* ON and HIGH cells, presumably because the pattern of binding sites and H3K27ac in the intervening region is similar in the two cell lines. The *Pax6*-DRR collision interval, however, is larger in Pax6 HIGH cells than ON cells probably because in *Pax6* HIGH there is a large region of disrupted chromatin (marked by H3K27ac) between these two sites.

The duration of promoter-enhancer interactions (Supplementary Fig. 1c), is an order of magnitude smaller than the collision interval. The interaction duration is longest in the *Pax6* ON cells where there are more protein binding sites within the regulatory regions than in the other cell lines. The fact that the difference between, e.g., interaction durations in *Pax6* OFF and ON cells is at most about two-fold is perhaps surprising. This suggests that the stabilisation of loops by protein clusters is modest, and might be perturbed by varying, for instance, protein number and switching rate.

Collision intervals and interaction durations were also obtained from regions across the locus; we consider 10 kbp regions which tile the locus between *Rcn1* and *Elp4*, and perform the same mean collision interval and interaction duration measurements for every possible pair of regions in order to build up a map. These maps (Supplementary Fig. 1d) are reminiscent of Hi-C data, but instead show information on dynamics which would be inaccessible experimentally.

6 Effect of varying loop extrusion parameters

As detailed in the main text, we also varied some of the loop extrusion parameters in order to understand how these affect chromatin dynamics.

First, we varied the rate of loop extrusion $k_{\rm ex}$, considering values of $k_{\rm ex} = 1, 2$, and 4 bp τ^{-1}. Interestingly, the effect of changing this parameter is subtle. One might expect that increasing $k_{\rm ex}$ would increase the dynamics of chromatin loci. In fact, the effect on the mobility of chromatin beads varied across the locus: in some regions there was an increases, while in others there was a decrease (Supplementary Figs. 6a-b). We observed no clear connection with ATAC or H3K27ac properties, but in general beads close to CTCF sites (extruder loop anchors) tended to have a *decreased* mobility for larger $k_{\rm ex}$ (Supplementary Fig. 6c). This can be rationalised via the following observations. It is likely that for beads which are far from CTCF sites, increasing extruder rate does lead to an increase in mobility, but since an extruder moves past a given bead only occasionally during a simulation, any effect is modest. If the extrusion rate is larger, the extruder will travel further before unbinding, and will reach CTCF sites more quickly, stopping there for longer. This has two effects: first, when an extruder is halted at a CTCF, the site is held in a loop, which puts a constraint on its motion, reducing its mobility. Consistent with this, we found that the density near CTCF sites shows an increase if extruder rate is increased (Supplementary Fig. 6a). Since extruder mediated looping likely facilitates/stabilises protein mediated bridging between nearby ATAC sites there is also a second effect: if an increased extruder rate leads to an increase in CTCF looping, this will change which ATAC bridges are stabilised (i.e., making some ATAC-loops more likely, and some less likely). Since looping/protein bridging is a constraint on motion, this will lead to an increased mobility in some regions, and decreased mobility in others.

The second parameter varied was the number of CTCF sites. Specifically, we added new CTCF sites at random positions across the locus, increasing the total number of sites by 50% in each cell line. For the *Pax6* HIGH cells the total number of CTCF sites across the locus was increased from 30 to 44, while for ON cells the increase was from 28 to 42. In *Pax6* OFF cells there are fewer CTCF sites to start with, giving an increase from 9 to 13 sites across the locus. In all cases some of the new sites were

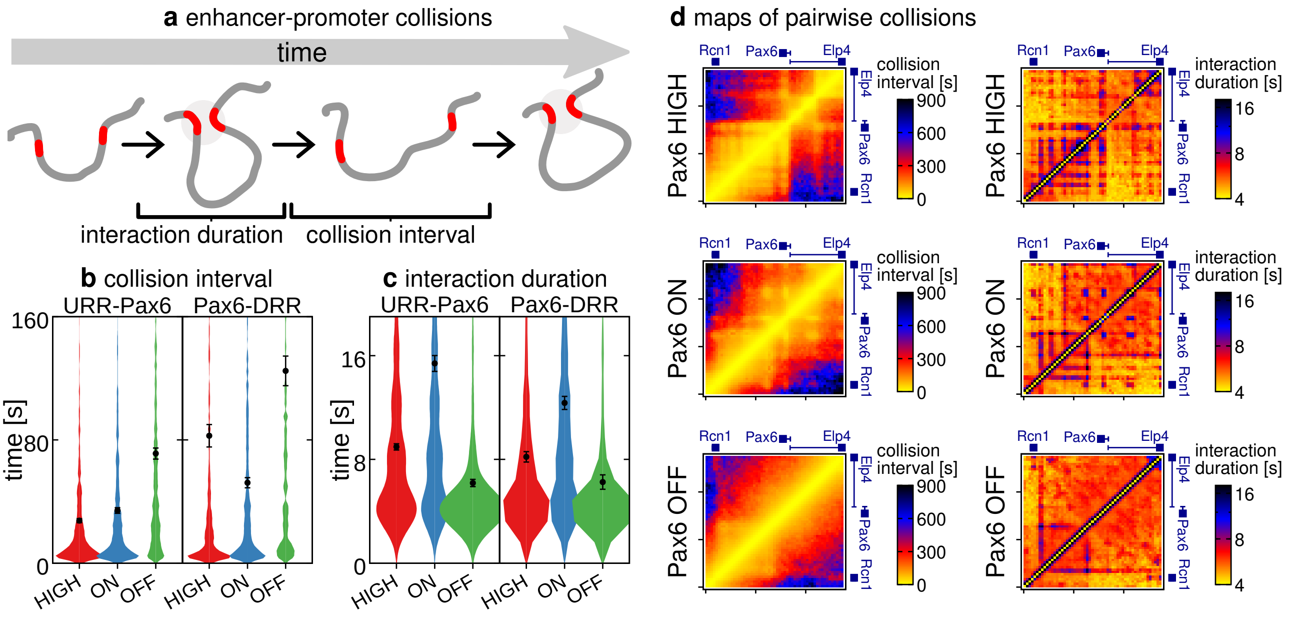

Supplementary Fig. 1 | Enhancer-promoter collisions. a. The time interval between successive promoter-enhancer interaction events, and the duration of such events can be measured. We considered a region covering the *Pax6* promoters (chr2:105,509,500-105,520,500 mm9 reference genome) and regions covering the upstream and downstream regulatory regions (chr2:105,444,500-105,457,500 and chr2:105,629,500-105,640,500 respectively), defining a collision as when the centre of mass of the two regions has a separation of $6\sigma \approx 106$ nm or less (see Supplementary Information for details). **b.** Plot showing the collision interval for each enhancer in the three cell lines. Points and error bars show the mean and standard error in the mean (SEM); were error bars are not visible, they are smaller than the points. The full distribution is represented as a violin plot; from left to right, the numbers of measurements making up each distribution are $n = 906, 668, 428$ and $n = 365, 513, 253$. **c.** Similar plots showing the mean duration of interaction (collision) events. Again, points and error bars show the mean and SEM, with the violin plots showing the full distribution. From left to right, the numbers of measurements making up each distribution are $n = 892, 662, 416$ and $n = 346, 505, 233$ **d.** Maps showing the mean collision interval (left) and mean interaction duration (right) for pairs of 10 kbp windows tiled across the locus.

within the region encompassing *Rnc1*, *Pax6*, and *Elp4*. While adding new CTCF sites in this way is somewhat artificial, and could not be replicated experimentally, it nevertheless provides insight into the effect of CTCF site density on dynamics (e.g., giving insight into the difference between a region rich in CTCF sites and a region poor in CTCF sites). Similar to the case of varying k_{ex}, adding new CTCF sites leads to a small increase in mobility in some regions, and a small decrease in others (Supplementary Figs. 6e-f). There is again no apparent dependence on ATAC or H3K27ac properties, but a significant effect is found at the new CTCF sites themselves. Specifically, there is a *decrease* in mobility in the vicinity of the new sites (by ∼5% on average). This can be explained by considering that CTCF sites are more likely to be involved in loops mediated by halted extruders, which pose a constraint on the motion (consistent with this we observed an increased local density at these sites, Supplementary Fig. 6e). Increasing the overall linear density of CTCF sites means that extruders are more likely to reach and stall at these sites (a similar effect to increasing the extrusion rate). As above, changing the pattern of CTCF looping will redistribute protein binding across the region, leading to small increases or decreases in mobility.

Intriguingly, varying these extrusion parameters did not affect the shape change parameter (Supplementary Figs. 6d,h). In other words, the rate at which the overall configuration of the locus, as defined by the relative positions of the *Pax6* promoter and its two enhancer regions, did not change. This result might be specific to this locus, as we previously showed that removing loop extruders did not have a significant effect on the interactions at *Pax6* [1]. For example, for a locus with a promoter and enhancer at opposite ends of a CTCF loop domain, one might expect loop extrusion to play a more significant role in driving promoter-enhancer interactions, and varying the extruder properties may well have a larger effect.

Overall these results show that the effects of varying loop extrusion parameters are subtle and difficult to predict *a priori* (i.e., without performing the simulation). This is because all of the factors affecting dynamics work in concert: the precise patterns of ATAC sites, CTCF sites and H3K27ac regions. Varying loop extrusion parameters affects the way these different factors work together, for example leading to a redistribution of protein binding at ATAC sites across the locus.

7 Two models for simulating alpha amanitin treatment

As detailed in the main text, we considered two alternative scenarios for including alpha amanitin treatment in simulations.

In Model 1 (Fig. 5c) all proteins were removed from the system, as these represent complexes of transcription factors and RNA polymerase. The simulation was then run for $1.5 \times 10^6\,\tau$, with loop extrusion continuing. Only configurations from the final $8 \times 10^5\,\tau$ of the simulation were used to generate plots (Figs. 5d,g-i) to allow for a relaxation after the simulated ‘treatment’. Six independent simulations, with a total of 20 copies of each locus for each cell line were performed.

In Model 2 (Fig. 5e) protein-chromatin bonds were instead made permanent at the point of treatment. We first ran a standard simulation for $1.5 \times 10^6\, \tau$. Then to simulate the treatment we identify all proteins which are within a distance $1.8\,\sigma$ of a chromatin bead; a new harmonic bond was then added between these protein and the chromatin beads

$$U_{AA}(r_{i,j}) = U_{WCA}(r_{i,j}) + K_{AA}(r_{i,j} - r_0)^2$$

with $K_{AA} = 20 k_B T/\sigma^2$ and $r_0 = 1.1.\sigma$. At the same time, all other protein-chromatin attractions were switched off. The simulation was then continued for a further $1.5 \times 10^6\, \tau$, with loop extrusion continuing. Again, only configurations from the final $8 \times 10^5\, \tau$ of the simulation were used to generate plots (Figs. 5f-i) to allow for a relaxation after the simulated treatment.

References

1. A. Buckle, et al. *Polymer Simulations of Heteromorphic Chromatin Predict the 3-D Folding of Complex Genomic Loci*. Molecular Cell **72**, 786 (2018).
2. D. Rico, et al. *High-resolution simulations of chromatin folding at genomic rearrangements in malignant B-cells provide mechanistic insights on proto-oncogene deregulation*. bioRxiv 2021.03.12.434963 (2021).
3. M. Chiang, et al. *Predictive Polymer Models for 3D Chromosome Organization.* In: Bicciato, S., Ferrari, F. (eds) Hi-C Data Analysis. Methods in Molecular Biology, 2301. Humana, New York, NY. (2022).
4. M. Chiang, et al. *Gene structure heterogeneity drives transcription noise within human chromosomes*. bioRxiv 2022.06.09.495447 (2022).
5. C. A. Brackey, D. Marenduzzo, and N. Gilbert. *Mechanistic modeling of chromatin folding to understand function.* Nature Methods **17**, 767 (2020).
6. C. A. Brackley, et al. *Simulated binding of transcription factors to active and inactive regions folds human chromosomes into loops, rosettes and topological domains*. Nucleic Acids Research **44**, 3503 (2016).
7. C. A. Brackley, et al. *Predicting the three-dimensional folding of cis-regulatory regions in mammalian genomes using bioinformatic data and polymer models*. Genome Biology **17**, 59 (2016).
8. C. A. Brackley, et al. *Ephemeral protein binding to DNA shapes stable nuclear bodies and chromatin domains*. Biophysical Journal **112**, 1085 (2017).
9. A.-M. Florescu, P. Therizols, and A. Rosa. *Large Scale Chromosome Folding Is Stable against Local Changes in Chromatin Structure*. PLOS Computational Biology **12**, 1 (2016).
10. G. Fudenberg, et al. *Formation of Chromosomal Domains by Loop Extrusion*. Cell Reports **15**, 2038 (2016).
11. A. Goloborodko, J. Marko, and L. Mirny. *Chromosome compaction via active loop extrusion*. Biophysical Journal **110**, 2162 (2016).
12. S. Plimpton. *Fast Parallel Algorithms for Short-Range Molecular Dynamics*. Journal of Computational Physics **117**, 1 (1995).
13. E. J. Banigan and L. A. Mirny. *Loop extrusion: theory meets single-molecule experiments*. Current Opinion in Cell Biology **64**, 124 (2020). Cell Nucleus.
14. A. Rosa and R. Everaers. *Structure and Dynamics of Interphase Chromosomes*. PLOS Computational Biology **4** (2008).
15. L. A. Mirny. *The fractal globule as a model of chromatin architecture in the cell*. Chromosome Research **19**, 37 (2011).
16. H. Hajjoul, et al. *High-throughput chromatin motion tracking in living yeast reveals the flexibility of the fiber throughout the genome*. Genome Research **23**, 1829 (2013).
17. S. Shinkai, et al. *Dynamic Nucleosome Movement Provides Structural Information of Topological Chromatin Domains in Living Human Cells*. PLOS Computational Biology **12**, 1 (2016).
18. J. Duan, et al. *Live imaging and tracking of genome regions in CRISPR/dCas9 knock-in mice*. Genome Biology **19**, 192 (2018).
19. L. Chang, et al. *Nuclear peripheral chromatin-lamin B1 interaction is required for global integrity of chromatin architecture and dynamics in human cells*. Protein & cell **13**, 258 (2022).
20. B. Langmead and S. L. Salzberg. *Fast gapped-read alignment with Bowtie 2*. Nature Methods **9**, 357 (2012).
21. A. R. Quinlan and I. M. Hall. *BEDTools: a flexible suite of utilities for comparing genomic features*. Bioinformatics **26**, 841 (2010).
22. Y. Zhang, et al. *Model-based Analysis of ChIP-Seq (MACS)*. Genome Biology **9**, R137 (2008).
23. J. Toedling, et al. *Ringo: an R/Bioconductor package for analyzing ChIP-chip readouts*. BMC Bioinformatics p. 221 (2007).
24. O. Fornes, et al. *JASPAR 2020: update of the open-access database of transcription factor binding profiles*. Nucleic Acids Research **48**, D87 (2019).
25. T. L. Bailey, et al. *MEME Suite: tools for motif discovery and searching*. Nucleic Acids Research **37**, W202 (2009).
26. A. S. Hansen, et al. *CTCF and cohesin regulate chromatin loop stability with distinct dynamics*. eLife **6**, e25776 (2017).
27. A. Buckle, et al. *capC-MAP: software for analysis of Capture-C data*. Bioinformatics **35**, 4773 (2019).
28. B. Langmead, et al. *Ultrafast and memory-efficient alignment of short DNA sequences to the human genome*. Genome Biology **10**, R25 (2009).
29. P. Danecek, et al. *Twelve years of SAMtools and BCFtools*. GigaScience **10** (2021).
30. M. Martin. *Cutadapt removes adapter sequences from high-throughput sequencing reads*. EMBnet.journal **17** (2011).
31. MATLAB. *version 9.8.0.1380330 (R2020a)*, (The MathWorks Inc., Natick, Massachusetts2020).
32. M. Doi and S. F. Edwards. *The Theory of Polymer Dynamics*, (Oxford University Press, Oxford, UK 1986).
