# Supplementary material for: Transcription modulates chromatin dynamics and locus configuration sampling
Source: Nat Struct Mol Biol. 2023 Aug 3;30(9):1275–85. doi: 10.1038/s41594-023-01059-8 (PMC10497412; doi:10.1038/s41594-023-01059-8)
Supplement: Supplementary file 2 — Reporting Summary [file 41594_2023_1059_MOESM2_ESM.pdf]

Corresponding author(s): Chris A Brackley; Nick GIlbert

Last updated by author(s): Jun 26, 2023

Reporting Summary

Nature Portfolio wishes to improve the reproducibility of the work that we publish. This form provides structure for consistency and transparency in reporting. For further information on Nature Portfolio policies, see our Editorial Policies and the Editorial Policy Checklist.

Statistics

For all statistical analyses, confirm that the following items are present in the figure legend, table legend, main text, or Methods section.

n/a	Confirmed	
☐	☒	The exact sample size (*n*) for each experimental group/condition, given as a discrete number and unit of measurement
☒	☐	A statement on whether measurements were taken from distinct samples or whether the same sample was measured repeatedly
☐	☒	The statistical test(s) used AND whether they are one- or two-sided<br>*Only common tests should be described solely by name; describe more complex techniques in the Methods section.*
☒	☐	A description of all covariates tested
☒	☐	A description of any assumptions or corrections, such as tests of normality and adjustment for multiple comparisons
☐	☒	A full description of the statistical parameters including central tendency (e.g. means) or other basic estimates (e.g. regression coefficient) AND variation (e.g. standard deviation) or associated estimates of uncertainty (e.g. confidence intervals)
☐	☒	For null hypothesis testing, the test statistic (e.g. *F*, *t*, *r*) with confidence intervals, effect sizes, degrees of freedom and *P* value noted<br>*Give P values as exact values whenever suitable.*
☒	☐	For Bayesian analysis, information on the choice of priors and Markov chain Monte Carlo settings
☒	☐	For hierarchical and complex designs, identification of the appropriate level for tests and full reporting of outcomes
☐	☒	Estimates of effect sizes (e.g. Cohen's *d*, Pearson's *r*), indicating how they were calculated

Our web collection on statistics for biologists contains articles on many of the points above.

Software and code

Policy information about availability of computer code

Data collection	Simulations were performed using the LAMMPS molecular dynamics software (version 12Dec2018), and the HiP-HoP model published in Buckle et al Molecular Cell 2018. Image capture and analysis were done using scripts written for Iola Spectrum (Scanalytics).
Data analysis	CaptureC data was analyzed using the capC-MAP 1.1.3 software (which uses Bowtie1.1.1, samtools 1.3.1 and cutadapt 1.11). Previously published ATAC-seq data were analyzed using Trim Galore v0.6.5, Bowtie2 v2.4.2, MACS 2.1.1 and the Bedtools suit. Previously published ChIP-on-chip data were analyzed using the "Ringo" bioconductor tool. The MEME suit of tools was used for CTCF motif analysis. For FISH, images were collected from at least 50 randomly selected nuclei for each experiment and then analysed using Iola scripts that calculate the distance between two probe signals. Custom scripts used for analysis of simulation data have been deposited in the Edinburgh DataShare digital repository and are accessible at DOI:10.7488/ds/7477 (https://doi.org/10.7488/ds/7477).

For manuscripts utilizing custom algorithms or software that are central to the research but not yet described in published literature, software must be made available to editors and reviewers. We strongly encourage code deposition in a community repository (e.g. GitHub). See the Nature Portfolio guidelines for submitting code & software for further information.

Data

Policy information about availability of data

All manuscripts must include a data availability statement. This statement should provide the following information, where applicable:

- Accession codes, unique identifiers, or web links for publicly available datasets
- A description of any restrictions on data availability
- For clinical datasets or third party data, please ensure that the statement adheres to our policy

Previously published sequencing data was obtained from the NCBI Gene Expression Omnibus (GEO) as follows: ATAC-seq data GSE119656; ChIP-on-chip data

GSE119659, GSE119658 and GSE120665; CaptureC data GSE120666. New sequencing data (NG CaptureC experiment) generated in this study have been deposited in the GEO under accession numbers GSE235334 and GSE235335. New FISH data and simulation data generated for this study, together with processed data underlying all figures, have been deposited in the Edinburgh DataShare digital repository and are accessible at DOI:10.7488/ds/7477 (https://doi.org/10.7488/ds/7477). Sequencing data were aligned to the mouse mm9 build reference genome which is available from the UCSC Genome Browser website (https://hgdownload.soe.ucsc.edu/goldenPath/mm9/bigZips/mm9.2bit).

Field-specific reporting

Please select the one below that is the best fit for your research. If you are not sure, read the appropriate sections before making your selection.

☒ Life sciences ☐ Behavioural & social sciences ☐ Ecological, evolutionary & environmental sciences

For a reference copy of the document with all sections, see nature.com/documents/nr-reporting-summary-flat.pdf

Life sciences study design

All studies must disclose on these points even when the disclosure is negative.

Sample size	For FISH at least 50 nuclei were analysed per sample. This was number was determined from a power calculation to enable true differences to be identified. Also see Naughton et al., 2013 NSMB
Data exclusions	No data was excluded
Replication	Two biological replicated were generated for all NG CaptureC experiments. Individual replicates were found to be consistent, and for paper figures were combined.
Randomization	Randomization was not applicable as there was no grouping in this study.
Blinding	The study did not involve human individuals or animals, and no subjective evaluations were performed. Blinding was therefore not applicable in this study.

Reporting for specific materials, systems and methods

We require information from authors about some types of materials, experimental systems and methods used in many studies. Here, indicate whether each material, system or method listed is relevant to your study. If you are not sure if a list item applies to your research, read the appropriate section before selecting a response.

Materials & experimental systems

n/a	Involved in the study	
☒	☐	Antibodies
☐	☒	Eukaryotic cell lines
☒	☐	Palaeontology and archaeology
☒	☐	Animals and other organisms
☒	☐	Human research participants
☒	☐	Clinical data
☒	☐	Dual use research of concern

Methods

n/a	Involved in the study	
☒	☐	ChIP-seq
☒	☐	Flow cytometry
☒	☐	MRI-based neuroimaging

Eukaryotic cell lines

Policy information about cell lines

Cell line source(s)	b-TC3 cells (referred to as Pax6-HIGH cells in the paper) were used in this study, obtained from DSMZ (Cat# ACC-324, RRID: CVCL_0172). They have not been authenticated but have been used in many of our previous studies e.g. Buckle, Brackley et al., 2018 Molecular Cell
Authentication	None
Mycoplasma contamination	Cell lines are routinely tested for Mycoplasma by technical services within the Institute Genetics and Cancer; the cell line used int the study tested negative.
Commonly misidentified lines (See ICLAC register)	No commonly misidentified cell lines were used in this study.
